# Histidine-Tagged Folate-Targeted Gold Nanoparticles for Enhanced Transgene Expression in Breast Cancer Cells In Vitro

**DOI:** 10.3390/pharmaceutics14010053

**Published:** 2021-12-27

**Authors:** Calrin Joseph, Aliscia Daniels, Sooboo Singh, Moganavelli Singh

**Affiliations:** 1 Nano-Gene and Drug Delivery Group, Discipline of Biochemistry, School of Life Sciences, College of Agriculture, Engineering and Science, University of KwaZulu-Natal, Private Bag X54001, Durban 4000, South Africa; joseph.calrin3@gmail.com (C.J.); DanielsA@ukzn.ac.za (A.D.); 2 School of Chemistry and Physics, University of KwaZulu-Natal, Private Bag X54001, Durban 4000, South Africa; singhso@ukzn.ac.za

**Keywords:** gene delivery, gold nanoparticles, breast cancer, histidine, folate receptor, targeting

## Abstract

Nanotechnology has emerged as a promising treatment strategy in gene therapy, especially against diseases such as cancer. Gold nanoparticles (AuNPs) are regarded as favorable gene delivery vehicles due to their low toxicity, ease of synthesis and ability to be functionalized. This study aimed to prepare functionalized AuNPs (FAuNPs) and evaluate their folate-targeted and nontargeted pCMV-*Luc*-DNA delivery in breast cancer cells in vitro. CS was added to induce stability and positive charges to the AuNPs (Au-CS), histidine (Au-CS-His) to enhance endosomal escape and folic acid for folate-receptor targeting (Au-CS-FA-His). The FAuNP:pDNA nanocomplexes possessed favorable sizes (<135 nm) and zeta potentials (<−20 mV), strong compaction efficiency and were capable of pDNA protection against nuclease degradation. These nanocomplexes showed minimal cytotoxicity (>73% cell viability) and enhanced transgene activity. The influence of His was notable in the HER2 overexpressing SKBR3 cells, which produced higher gene expression. Furthermore, the FA-targeted nanocomplexes enhanced receptor-mediated endocytosis, especially in MCF-7 cells, as confirmed by the receptor competition assay. While the role of His may need further optimization, the results achieved suggest that these FAuNPs may be suitable gene delivery vehicles for breast cancer therapeutics.

## 1. Introduction

Breast cancer is recognized as the most common cancer among women worldwide [1], with disturbingly high mortality rates in the African continent [2]. Due to cancer being one of the most prevalent pandemics that the world faces, scientists are continuously trying to find the most effective form of treatment. Conventional therapies such as chemotherapy, radiation and surgery are commonly used with the hope of saving as many lives as possible but are limited by their health concerns. The inability of toxic chemotherapeutic drugs to selectively target cancer cells can result in adverse effects ranging from hair loss to infertility [3,4], while surgery and radiation can result in wound infections [5] and pulmonary complications [6]. The effectiveness of these treatments has also come into question due to the reoccurrence of certain cancers following treatment [7,8]. Gene therapy has since its inception grown in popularity as an alternative form of treatment that addresses the shortcomings of these aggressive treatments. However, its success is largely dependent on the vector chosen for delivery. Although viral delivery vectors have been the most utilized thus far, their associated safety, immunogenicity and production challenges have fueled the search for non-viral modalities capable of safe and efficient and targeted gene delivery [9].

The emergence of nanoparticles (NPs) in the clinical field, known as nanomedicine, has revolutionized diagnostics and therapeutics. Gold nanoparticles (AuNPs), with their favorable physiochemical properties, have progressed into one of the most popular and versatile NPs for application in gene delivery. This metallic NP is renowned for its ease of synthesis, tailorable size, easy functionalization, low toxicity, unique optical properties and biocompatibility [10]. AuNPs possess the ability to be easily functionalized, leading to further possible advantages for gene delivery, such as the appending of cell-specific targeting moieties. Furthermore, the surface modification of these NPs can increase circulation, reduce cytotoxicity, increase cellular uptake and allow for easy attachment of therapeutic nucleic acid molecules onto the AuNP surface [11,12].

Chitosan (CS) is a natural, nontoxic, biodegradable and biocompatible polysaccharide derivative of chitin produced by its partial deacetylation and is sourced from the exoskeleton of crustaceans [13]. CS has also evolved into a popular coating agent due to the interaction between their active amino groups and the negatively charged metal NPs [14]. In diluted acidic aqueous solutions (pH < 6), the amino groups are protonated and improve solubility [15], as well as providing CS with high cationic potential and a strong affinity for negatively charged nucleic acids, allowing for ionic complex formation [16,17].

Histidine (His) is a polar amino acid with an aromatic nitrogen and an imidazole ring side chain [18] and is considered versatile with molecular interactions attributed to its unique structure [19]. His is recognized as one of the strongest binders to metal ions such as Au, Ag and Zn, due to the three main groups found within His, viz., the carboxylic, amino and imidazole groups [20]. Several studies have to date investigated this interaction [21,22]. Functionalization with His provides a possibility of enhanced therapeutic delivery due to the presence of the imidazole ring, which influences the disruption and destabilization of cell and endosomal membranes [23], thereby improving the delivery of therapeutic agents. The use of the imidazole-containing compounds such as histidine with buffering capacity within the endosomal pH range has been utilized to effect the escape of therapeutic nucleic acid cargo from the endosome in a phenomenon referred to as the “proton sponge effect” [24,25].

The anatomical and pathophysiological differences between tumors and healthy tissues can be exploited due to the higher expression of molecules, such as receptors, on tumor cells than on normal healthy cells [26]. The ability of tumors to attract NPs allows for active targeting and site-specific targeted delivery of the therapeutic molecule. For the process of active targeting, a specific targeting moiety or ligand must be conjugated to the NP surface. This exploits the expression or overexpression of receptors on cancer cells, allowing for the targeting ligand to bind to their cognate receptors for cellular uptake [27]. The folate receptor (FR) has emerged as a useful target for the tumor-specific delivery of genes and drugs due to their overexpression in a wide range of human cancers, such as breast cancer [28]. A high degree of specificity arises due to the lack of FR expression in nonproliferating healthy cells [29]. FA is an attractive ligand for NP targeting of FRs, due to the distinctive characteristics it possesses. It is a low MW (441 Da) ligand that is nonimmunogenic, with a carboxylic acid functional group that allows for simple conjugation chemistry [30,31].

This study addresses the need for an efficient gene delivery vehicle through a proof of principle investigation that utilizes gold nanoparticles (AuNPs) functionalized with CS, His and FA to deliver plasmid pCMV-*Luc*-DNA (pDNA) to breast cancer cells in vitro. CS confers positive charges and stability on the AuNPs, while His imbues “proton sponge” capabilities that can enhance the endosomal escape. Furthermore, the conjugation of the FA ligand was introduced to target the overexpressed FRs, providing tumor-specific uptake by the breast cancer cells.

## 2. Materials and Methods

### 2.1. Materials

Gold (III) chloride (HAuCl_4_), chitosan (190-310 kDa, >75% deacetylated), DL-histidine (His, MW 155.15 g/mol), folic acid, 1-ethyl-3-(3-dimethyl aminopropyl) carbodiimide (EDC), N-hydroxysuccinimide (NHS), dialysis tubing (MWCO = 12 kDa) and the bicinchoninic acid (BCA) kit were purchased from Sigma-Aldrich, St. Louis, MO, USA. 3-[4,5-dimethylthiazol-2-yl]-2.5-diphenyltetrazolium bromide (MTT), tris (hydroxymethyl) aminomethane, acetic acid, ethylenediaminetetraacetic acid (EDTA) disodium salt, trisodium citrate, 2-[4-(2-hydroxyethyl)piperazin-1-yl]ethanesulfonic acid (HEPES), sodium dodecyl sulphate (SDS), acridine orange (AO), ethidium bromide (EB), and phosphate-buffered saline (PBS) tablets were obtained from Merck, Darmstadt, Germany. pCMV-*Luc*-pDNA was supplied by the Plasmid Factory, Bielefeld, Germany. Agarose (ultrapure) was supplied by Bio-Rad Laboratories, Inc., Hercules, CA, USA. Tissue culture reagents (Eagles Minimum Essential Medium (EMEM), trypsin-EDTA and antibiotic (penicillin (5000 units/mL)/streptomycin (5000 µg/mL)) were sourced from Lonza BioWhittaker, Walkersville, MD, USA. Gamma-irradiated fetal bovine serum (FBS) was sourced from Hyclone GE Healthcare (Logan, UT, USA). The tissue culture plasticware was either procured from Nest Biotechnologies (Wuxi, China) or Corning Incorporated (New York, NY, USA). The human embryonic kidney (HEK293) and breast adenocarcinoma cell lines MCF-7 and SKBR3 were originally supplied by the American Type Culture Collection, Manassas, VA, USA. The commercial transfection reagent Lipofectin^®^ was obtained from Thermo Fisher Scientific, Waltham, MA, USA. The luciferase assay and the cell lysis reagents were obtained from the Promega Corporation, Madison, WI, USA. All experiments utilized 18-MOhm water (Milli-Q, Millipore, Molsheim, France). All other reagents were of analytical grade and sourced locally.

### 2.2. Gold Nanoparticle Synthesis

An adaption of the Turkevich trisodium citrate reduction method was employed to synthesize colloidal gold nanoparticles (AuNPs) from HAuCl_4_ [32]. Approximately 375 µL of HAuCl_4_ (30 mM) was added to 18-MOhm water (30 mL) that was brought to a temperature of 85 °C. After that, 1 mL of 1% (*w*/*v*) trisodium citrate was added, with a gradual color change from dark purple to a deep wine red being observed. The solution was heated for a further 5 min, then cooled and stored in the dark at room temperature.

### 2.3. Synthesis of Chitosan Functionalized Gold Nanoparticles

Chitosan (CS)-functionalized AuNPs (Au-CS) were prepared by the dropwise addition of approximately 4 mL of the synthesized AuNPs to 1 mg of CS solution (0.5 mg/mL in 1% (*v*/*v*) acetic acid) under constant stirring [33] for 24 h. The Au-CS solution was dialyzed (MWCO 12 kDa) for 2 h against 18-MOhm water to remove any unbound CS.

### 2.4. Synthesis of Chitosan and Histidine Functionalized Gold Nanoparticles

AuNPs functionalized with histidine (His) and CS (Au-CS-His) were prepared from the Au-CS NPs (Section 2.2). Briefly, 5-mL His (100 µM) was added dropwise to the Au-CS NPs [34]. After an hour of constant stirring, the resulting Au-CS-His solution was dialyzed as in Section 2.2 over 24 h in 18-MOhm water to remove any unbound His.

### 2.5. Synthesis of Chitosan, Histidine and Folate Functionalized Gold Nanoparticles

Folate (FA) (1 mg/mL) was prepared by dissolving 5-mg folic acid, 15-mg EDC and 12-mg NHS in 5 mL of 18-MOhm water. The pH was adjusted to 7.4, and the solution was stirred over 24 h. To this FA solution was added CS with stirring for 24 h to obtain a 4:1 (CS:FA) ratio (*v*/*v*). Thereafter, 4 mL of the synthesized AuNPs (Section 2.2) were added dropwise with stirring to 2 mL of the CS-FA solution. The Au-CS-FA solution was then stirred for 24 h. About 5 mL of 100-µM His was added dropwise with stirring to the Au-CS-FA solution. Following each conjugation step for Au-CS-FA synthesis, dialysis was conducted as in Section 2.2.

### 2.6. Preparation of Nanocomplexes 

To formulate the nanocomplexes, increasing amounts of the respective functionalized AuNP (FAuNP) were added to a fixed amount of pCMV-*Luc*-DNA (pDNA, 0.25 µg/µL) and made up to a volume of 10 µL with HEPES-buffered saline (HBS) (20-mM HEPES and 150-mM NaCl, pH 7.4). Nanocomplexes were thereafter incubated at room temperature for 1 h.

### 2.7. Nanoparticle and Nanocomplex Characterization

The absorption spectra of the AuNPs and FAuNPs were determined using UV–Vis spectrophotometric analysis (Jasco V-730 Bio Spectrophotometer, JASCO Corporation, Hachioji, Japan) in a wavelength range of 400–650 nm.

Nanoparticle tracking analysis (NTA) (Nanosight NS500, Malvern Instruments, Worcestershire, UK) was used to evaluate the hydrodynamic sizes, zeta potentials and polydispersities of the NPs and nanocomplexes. The samples were first diluted (1:100, 1 mL) in 18-MOhm water. The optimum binding ratio of the nanocomplexes (obtained from Section 2.7) was assessed.

The morphology of the NPs and nanocomplexes was determined using a high-resolution transmission electron microscope (HRTEM). The NP or nanocomplex (5 µL) was added to a carbon-coated copper grid (400-mesh; Ted Pella Inc. Redding, CA, USA) and air-dried. The samples were observed using a JEOL JEM-2100 HRTEM (JEOL, Tokyo, Japan). Images were captured and analyzed using the MegaView III Soft Imaging Systems (SIS) and a side-mounted 3-megapixel digital camera (Olympus, Tokyo, Japan).

Fourier-transform infrared (FTIR) spectroscopy was conducted using a Perkin Elmer Spectrum 100 FTIR spectrometer fitted with a Universal Attenuated Total Reflectance (ATR) component in the wavelength range of 4000–400 cm^−1^. NP samples were placed onto the ATR crystal, and the FTIR spectral data was plotted using the associated Origin 6.0 software.

### 2.8. Band Shift Assay

The band shift assay [35] was utilized to assess the optimal binding ratios (*w*/*w*) of the pDNA to the FAuNPs. Nanocomplexes were formulated as in Section 2.5. A positive control containing pDNA (0.25 µg/µL) only was used to visualize the migration of unbound pDNA. Following complex formation, gel loading buffer (40% sucrose and 0.25% bromophenol blue) was added. The nanocomplexes were then subjected to agarose gel electrophoresis on 1% (*w*/*v*) agarose gel containing 2 µL of ethidium bromide (EB, 10 mg/mL) at 50 V for 90 min using a Bio-Rad Mini-Sub^®^ electrophoretic apparatus (Richmond, VA, USA). The final gel was viewed, and images were captured using a Vacutec Syngene G: Box BioImaging system (Syngene, Cambridge, UK).

### 2.9. Ethidium Bromide Intercalation Assay

The ethidium bromide (EB) intercalation assay [35] was used to assess the ability of FAuNPs to compact the pDNA. Approximately 100 µL of HBS and 2 µL of EB (100 µg/mL) was added to a 96-well FluorTrac flat-bottomed black plate (Greiner Bio-One, Frickenhausen, Germany). Using a GloMax^®^-Multi Detection System (Promega BioSystems, Sunnyvale, CA, USA), a baseline fluorescence of 0% was established by measuring the fluorescence value at excitation and emission wavelengths of 520 nm and 610 nm, respectively. Thereafter, 1.2 μg of pDNA was added to the mixture, and the measured fluorescence was set as 100%. The FAuNPs (1-µL aliquots) were then added, and the fluorescence was measured until a plateau in the readings was reached. The relative fluorescence (F_r_) was calculated as in Equation (1):

(%) F_R_ = ((F_i_ − F_0_)/(F_max_ − F_0_)) × 100 (1)

F_i_ is the fluorescence reading after adding each FAuNP increment, F_0_ is the fluorescence reading of EB alone (no pDNA) and F_max_ is the fluorescence reading resulting from the EB–DNA interaction. The relative fluorescence F_R_ (%) was then plotted against the incremental masses (μg) of the FAuNPs.

### 2.10. Nuclease Protection Assay

The degree of protection against nuclease digestion afforded by the FAuNPs to the pDNA was evaluated using the nuclease protection assay [35]. Nanocomplexes at the suboptimum, optimum and supraoptimum ratios (*w*/*w*), as determined from the band shift assay (Section 2.2, Table 1), were treated with FBS to a final concentration of 10% (1 µL) and incubated at 37 °C for 4 h. Thereafter, 1.1 µL of EDTA (10 mM) was added to each nanocomplex to stop the action of the nucleases. Thereafter, 1.33 µL of 5% (*w*/*v*) SDS was added to the nanocomplexes, which were then incubated for 20 min at 55 °C to ensure the release of the pDNA from the FAuNPs. A positive control (C1) containing naked pDNA with no FBS and a negative control (C2) containing FBS-treated pDNA were included. All nanocomplexes and both controls were subjected to agarose gel electrophoresis, as previously described in Section 2.7.

### 2.11. MTT Cell Viability Assay

Cells were trypsinized and seeded into 96-well plates at a density of 2 × 10^5^ cells/well and incubated at 37 °C overnight to allow for attachment. Thereafter, the growth medium (EMEM + 10% FBS + 1% antibiotics) was replaced. The nanocomplexes at the suboptimum, optimum and supraoptimum ratios (Table 1) were prepared in triplicate and added to their respective wells. A positive control was included, which was devoid of nanocomplexes and assumed to have 100% cell survival. The cells were then incubated for 48 h. Thereafter, the medium was removed and replaced with a 100-µL fresh serum-free medium containing 10-µL MTT reagent (5 mg/mL in PBS). Following a 4-h incubation at 37 °C, the medium containing MTT reagent was removed, and 100-uL DMSO was added to each well for solubilization of the formazan crystals. The absorbance was read at 570 nm in a Mindray MR-96A microplate reader (Vacutec, Hamburg, Germany), using DMSO as the blank.

### 2.12. Apoptosis Assay

Cells were seeded as in Section 2.10 and incubated at 37 °C for 24 h. The spent medium was replaced, and the nanocomplexes were added to the cells. The positive control utilized consisted of untreated cells. Cells were incubated for 48 h at 37 °C; after which the medium was aspirated, and the cells were washed with PBS and stained with the dual stain of acridine orange/ethidium bromide (AO/EB) (100-μg/mL AO and 100-μg/mL EB in PBS) for 5 min. The stained cells were observed under an Olympus fluorescence microscope and images captured using the associated CC12 fluorescence camera (Olympus Co., Tokyo, Japan). The apoptotic index was calculated as presented in Equation (2):

(2) $$\mathrm{Apoptotic}\;\mathrm{Index}=\frac{\mathrm{Number}\;\mathrm{of}\;\mathrm{Apoptotic}\;\mathrm{cells}}{\mathrm{Total}\;\mathrm{number}\;\mathrm{of}\;\mathrm{cells}\;\mathrm{counted}}$$

### 2.13. Luciferase Assay

Cells were seeded and treated as described in Section 2.10. Two sets of controls were included, one being the cell control with the absence of nanocomplexes and pDNA and the other a DNA control containing pDNA only. The control commercial transfection reagent Lipofectin^®^ was also complexed to pDNA according to the supplier’s protocols in triplicate. Lipofectin^®^ reagent (4, 6, 8 and 10 μg) and pCMV-*Luc* DNA (1 μg) were separately diluted in 25-μL serum-free EMEM and allowed to stand at room temperature for 30 min and then combined and incubated for 15 min at room temperature. The Lipofectin^®^:pDNA complexes were added to the cells according to the supplier’s transfection protocols, and the cells were incubated as for the other nanocomplexes for 48 h at 37 °C. After the 48-h incubation, the medium was removed, and the cells were washed twice with PBS. To each well, 50 µL of a 1 × cell lysis reagent was added, and the plate was shaken for 15 min. The cells were then scraped from the wells, and the cell suspensions were centrifuged in an Eppendorf 5415D centrifuge (Merck, Hamburg, Germany) at 12,000× *g* for 5 s. Approximately 20 µL of the cell-free supernatant in each tube was transferred to their respective wells of a white 96-well plate. Luminescence was measured in a GloMax^®^-Multi Detection System (Promega BioSystems, Sunnyvale, CA, USA), with 100 µL of the luciferase assay reagent automatically injected into each well. The protein content of the cell lysates was determined using the standard bicinchoninic acid (BCA) assay. The luciferase activity was normalized against the protein content, and the results were reported as relative light units (RLU) per mg of protein.

### 2.14. Competition Binding Assay

MCF-7 cells were chosen for this assay, as they are FR-rich and produce the best results for FA-targeted nanocomplexes. Cells were seeded as described in Section 2.10 and incubated at 37 °C for 24 h. The growth medium was replaced, and 25× excess FA (25 mg/mL) was added to the cells 30 min before adding the Au-Cs-FA-His nanocomplex. Thereafter, the cells were incubated at 37 °C for 48 h and then subjected to the luciferase assay as previously described in Section 2.13.

### 2.15. Statistical Analysis

The data were presented as the mean ± standard deviation (SD). The multiple group comparisons of the means were performed using two-way analysis of variance (ANOVA). This was followed by Tukey’s multiple comparisons test. The statistical significances were set at * *p* < 0.05, ** *p* < 0.01 and *** *p* < 0.001.

## 3. Results

### 3.1. Nanoparticle Synthesis and Characterization

**Figure 1:**
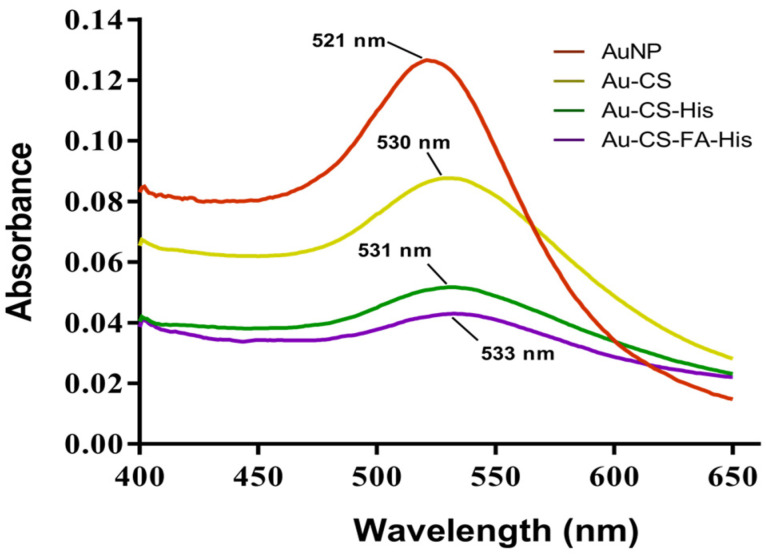
UV–Vis spectrum of AuNPs and FAuNPs.

The successful synthesis of the respective NPs was confirmed by the techniques employed. The UV–Vis spectra of the synthesized AuNPs and the FAuNPs are shown in Figure 1. The citrate-reduced AuNPs produced a distinct peak at the maximum absorbance wavelength (λmax) of 521 nm, which confirmed the presence of spherical AuNPs in solution and was consistent with the expected λmax of approximately 520 nm for spherical AuNPs [36]. Red shifts were observed for Au-CS, Au-CS-His and Au-CS-FA-His, with broader peaks in comparison to the AuNPs. Previous reports have indicated that the broadening of the peaks was due to the functionalization and attachment of ligands to metallic NPs [37,38].

**Figure 2:**
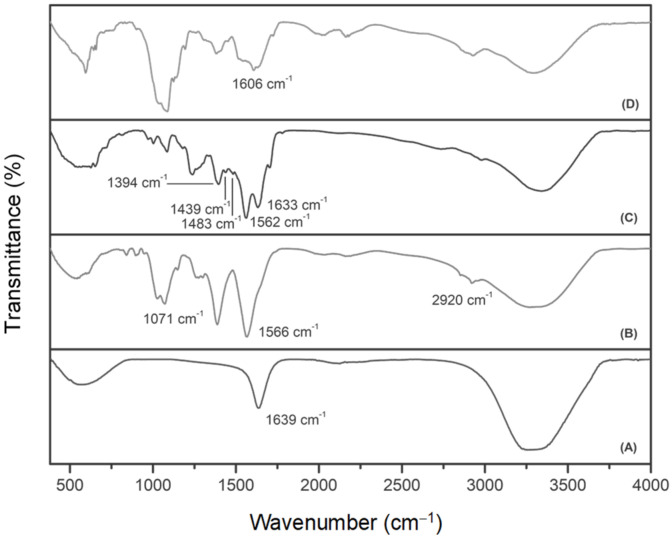
FTIR spectra of (**A**) AuNPs, (**B**) Au-CS, (**C**) Au-CS-His and (**D**) Au-CS-FA-His.

FTIR is a widely used characterization tool for the detection of functional groups. The FTIR spectra of AuNPs and FAuNPs are presented in Figure 2. Successful AuNP synthesis was confirmed by the presence of an asymmetric carboxylate stretching band at 1639 cm^−1^ (Figure 2A) [39,40]. Functionalization of AuNPs with CS was confirmed by the relevant peaks at 3267 cm^−1^ (N–H and O–H stretching), 2920 cm^−1^ (C–H symmetric stretching), 1566 cm^−1^ (N–H bending of the primary amine) and 1071 cm^−1^ (C–O stretching) (Figure 2B) [11,41,42]. These peaks also corresponded to the FTIR spectrum of CS (Supplementary Figure S1), with minor peak shifts and an intensity increase of the amine peak indicating successful binding to the AuNPs [42]. The confirmation of the addition of His was observed with peaks at ~1483 cm^−1^ (N-H symmetric bending of the amine), 1439 cm^−1^ (C–N stretching), 1633 cm^−1^ (C=C stretching) and 1394 cm^−1^ (COO– symmetric stretching) (Figure 2C) [43,44] and corresponded to the spectrum of His (Supplementary Figure S2). Furthermore, the peak at 1562 cm^−1^ is due to the imidazole group [45], thus confirming the presence of His. The preparation of Au-CS-FA-His was first confirmed by the conjugation of FA to CS with FTIR (Supplementary Figures S3 and S4). Due to the reaction between the carboxyl group of FA and the amide of CS, the peak of the carboxyl group at ~1686 cm^−1^ disappeared, while the peak of the amide shifted to ~1644 cm^−1^ and overlapped with the peak of the new C–N bond [33]. Further shifts were observed following the conjugation of the CS-FA and His to AuNPs (Figure 2D), with a characteristic peak at 1606 cm^−1^ due to the benzene ring of FA [46].

**Figure 3:**
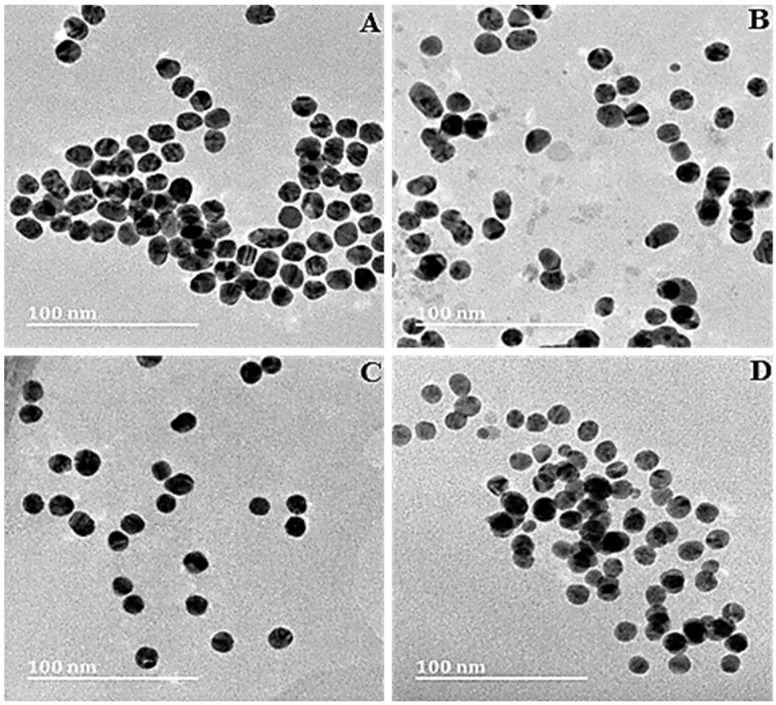
HRTEM images of (**A**) AuNPs, (**B**) Au-CS, (**C**) Au-CS-His and (**D**) Au-CS-FA-His. Scale bar = 100 nm.

High-resolution transmission electron microscopy (HRTEM) was utilized to visualize the ultrastructural characteristics of the NPs and are represented in Figure 3. The AuNPs and FAuNPs were all spherical, with Au-CS-FA-His showing evidence of slight aggregation (Figure 3D).

The nanoparticle tracking analysis (NTA) is a characterization technique that tracks the movement of individual particles in a solution to determine the average hydrodynamic diameter [47]. The sizes of the NPs and nanocomplexes are reflected in Table 2. The AuNPs were found to have a hydrodynamic diameter of 79.9 nm, and, following the functionlization of the AuNPs, an increase in size was observed. This increase is consistent with the red shifts in λmax observed by UV–Vis spectrophotometry. A change in NP size after surface modification is considered an indication of successful functionalization [48,49]. Furthermore, it was observed that the size of the nanocomplex increased compared to that of the unbound FAuNP, with the hydrodynamic diameters ranging between 116 nm and 127 nm. Overall, the hydrodynamic sizes of all nanocomplexes were larger than their respective uncomplexed NP.

The zeta (ζ) potential, which is predictive of the stability of NPs in a colloidal system, was also measured using the NTA. The ζ potential measurements shown in Table 2 revealed that AuNPs and FAuNPs were stable, with ζ potentials >20 mV. Irrespective of the nature of the charge (positive or negative), a magnitude of approximately 20 mV or higher suggests high stability [50]. Upon complexation with pDNA, the ζ potentials of all the FAuNPs changed from positive to negative and ranged between −21 mV and −35 mV, indicating high colloidal stability (Table 2) and confirmed the successful binding of the pDNA to the FAuNPs [51,52].

The polydispersity index (PDI) provides information on the size distribution of NPs. In general, indices lower than 0.1 are monodisperse and uniform in size, while indices as high as 0.4 indicate broad NP size dispersity, with a greater tendency of agglomeration [53]. All prepared NPs and their respective nanocomplexes exhibited low PDI values (most < 0.1), indicating their uniformity in size and distribution.

### 3.2. Band Shift Assay

**Figure 4:**
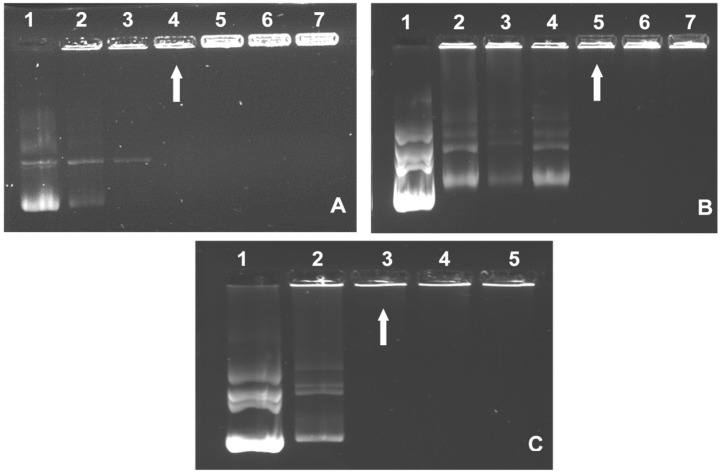
Band shift assays of nanocomplexes: (**A**) Au-CS (lanes 2–7: 0.25, 0.38, 0.5, 0.63, 0.75 and 0.88 µg); (**B**) Au-CS-His (lanes 2–7: 0.5, 0.6, 0.7, 0.8, 0.9 and 1.0 µg) and (**C**) Au-CS-FA-Hist (lanes 2–5: 1.13, 1.25, 1.38 and 1.5 µg). Lane 1: Control (0.25 µg pDNA). (**A**,**B**) (Lanes 2–7) and (**C**) (Lanes 2–5) show the respective pDNA nanocomplexes prepared using varying amounts of each FAuNP and a fixed amount of pDNA (0.25 µg). Arrows indicate complete binding.

The band shift or electrophoretic mobility shift assay was conducted to determine the minimum amount (µg) of FAuNPs required to completely bind a specific amount of pDNA. Through the electrostatic interaction between the positively charged FAuNPs and the negatively charged pDNA, favorable FAuNP:pDNA (nanocomplex) weight ratios were determined. The mass of each FAuNP was increased, while the pDNA (0.25 µg) mass remained constant until an electroneutral nanocomplex was formed. This electroneutral state is reached when the negative charge of the pDNA is completely neutralized by the positive charges of the FAuNPs. Therefore, due to this neutral charge, the nanocomplex will remain in the well instead of migrating into the agarose gel matrix. The agarose gel images are shown in Figure 4, with the resulting suboptimum, optimum and supraoptimum ratios reflected in Table 1. All FAuNPs were able to bind the pDNA at relatively low ratios. The conjugation of FA to CS in Au-CS-FA-His produced a higher optimum ratio (5:1) compared to its untargeted counterpart, Au-CS-His (3.2:1).

### 3.3. Dye Displacement Assay

**Figure 5:**
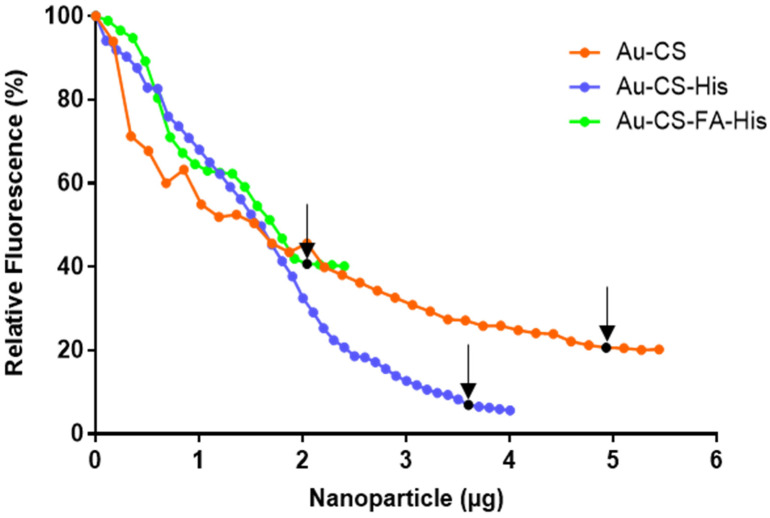
Ethidium bromide intercalation assay of Au-CS, Au-CS-His and Au-CS-FA-His. Black dots and arrows indicate the point of inflection.

EB is a fluorescent dye that can intercalate between the bases of DNA, causing an enhanced fluorescence of the dye [54]. The fluorescence of EB fully intercalated with pDNA was taken as 100% fluorescence. The incremental addition of the FAuNPs slowly caused a change in the conformation of the pDNA double-helix as it bound to the FAuNPs. Hence, the affinity for the dye was lost, resulting in a decay in fluorescence [55]. FAuNPs were added until the fluorescence readings reached a plateau, thus reaching the maximum compaction referred to as the point of inflection. The increasing fluorescence decay through the compaction of pDNA by the FAuNPs is presented in Figure 5.

It is evident that the FAuNPs could efficiently condense and compact the pDNA, albeit at varying degrees. All FAuNPs exhibited a fluorescence decay of more than 55%. Au-CS-FA-His displayed the lowest compaction ability (59.3% fluorescence decay), while Au-CS and Au-CS-His produced much higher compaction, with a 79.3% and 93% fluorescence decay, respectively.

### 3.4. Nuclease Protection Assay

**Figure 6:**
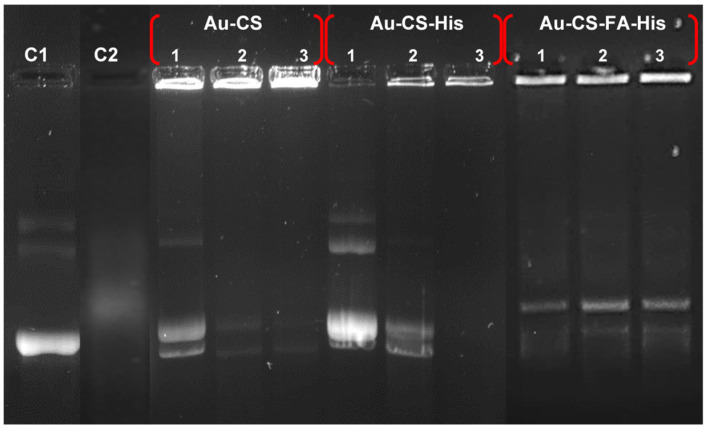
The nuclease protection assay of the nanocomplexes. Lane 1: positive control (untreated pDNA) and lane 2: negative control (pDNA digested with 10% FBS). Lanes 1: suboptimum ratio, 2: optimum ratio and 3: supraoptimum ratio for the indicated nanocomplex, as per Table 1.

Nanocomplexes at the suboptimum, optimum and supraoptimum ratios (Table 1) were tested, and the results are presented in Figure 6. The positive control (C1) contained untreated pDNA and showed three bands correlating to the circular, linear and supercoiled conformations. The negative control (C2) contained pDNA digested with 10% FBS, which showed complete degradation. All FAuNPs showed partial protection of the pDNA, with varied band intensities across the three ratios. The nuclease degradation of pDNA would most likely cause the supercoiled form to be nicked, resulting in relaxed circular and linear forms [56], which is evident in Figure 6. The supraoptimum ratios of Au-CS and Au-CS-His failed to produce any pDNA migration into the gel. However, the observed fluorescence in the wells suggested that the pDNA was still bound to the FAuNPs and was retained in the well.

### 3.5. MTT Cytotoxicity Assay

**Figure 7:**
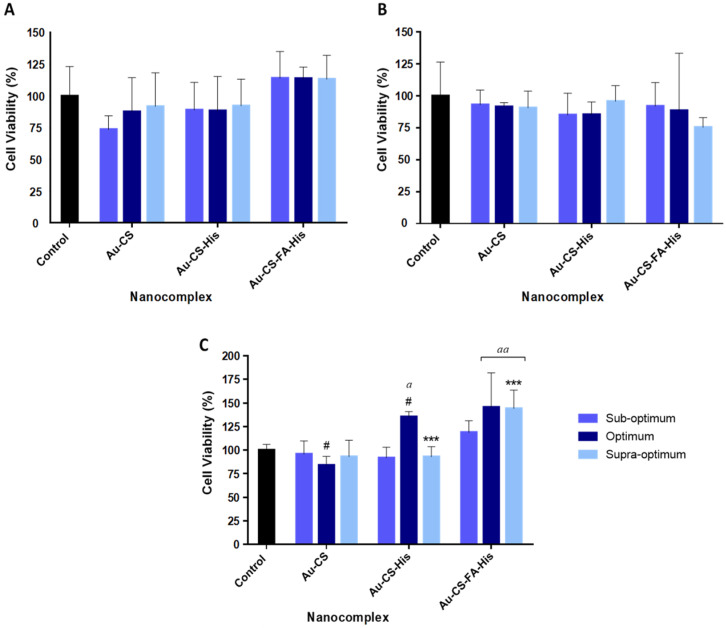
MTT cytotoxicity assay in the (**A**) HEK293, (**B**) SKBR3 and (**C**) MCF-7 cell lines. Data are represented as the mean ± SD (*n* = 3). *** *p* < 0.001 shows statistical significance between corresponding ratios of Au-CS-FA-His vs. Au-CS-His, ^#^ *p* <0.05 shows statistical significance between corresponding ratios of Au-CS-His vs. Au-CS and ^a^ *p* < 0.05 and ^aa^ *p* < 0.01 show statistical significance vs. control. The suboptimum, optimum and supraoptimum ratios are indicated in Table 1.

The cytotoxicity of the FAuNP nanocomplexes was evaluated in the HEK293, SKBR3 and MCF-7 cell lines using the MTT cytotoxicity assay. The MTT assay is a quantitative and colorimetric assay that measures cell viability. The principle of the assay is based on the conversion of the yellow salt 3-(4,5-dimethylthiazol-2-y1)-2,5-diphenyl tetrazolium bromide (MTT) into a dark blue/purple formazan product by the mitochondrial hydrogenases in living cells [57]. The resulting formazan product is directly proportional to the viable cell number but inversely proportional to the degree of cytotoxicity [57,58]. The results are expressed as the percentage of cell viability with respect to an untreated cell control (100%) and are presented in Figure 7.

All nanocomplexes at the suboptimum, optimum and supraoptimum ratios were well-tolerated, with cell viabilities over 73%. The FAuNP nanocomplexes showed minimal toxicity in the two human breast adenocarcinoma cell lines (MCF-7 and SKBR3 cell lines), with cell viabilities greater than 75%. However, Au-CS-His at the optimum ratio produced cell viability >100% in the MCF-7 cells. Furthermore, Au-CS-FA-His (Figure 7C) exhibited the least toxicity in the MCF-7 cells compared to Au-CS and Au-CS-His (Figure 7A,B). The cell viabilities at all three ratios of Au-CS-FA-His exceeded 100%, with the optimum and suboptimum ratios showing a significant increase compared to the control group (*p* < 0.01).

### 3.6. Apoptosis Assay

**Figure 8:**
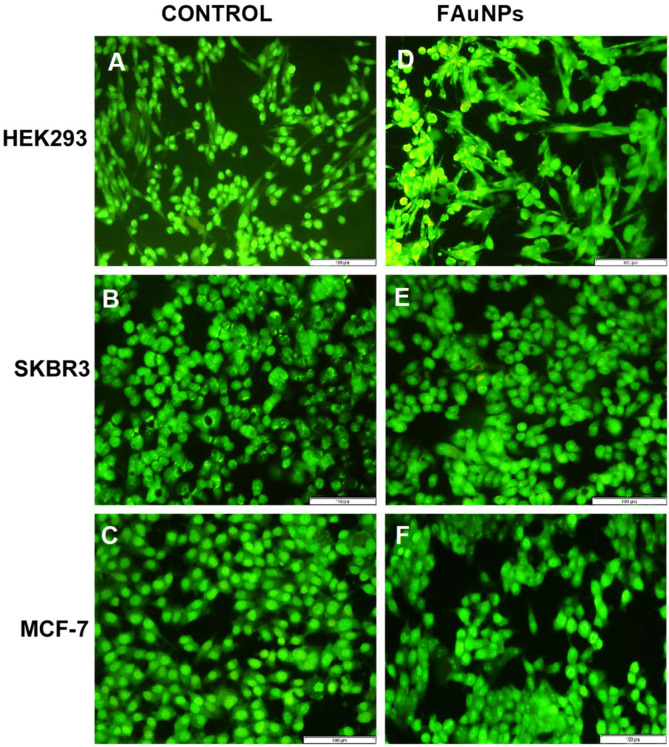
Fluorescent images from the acridine orange/ethidium bromide apoptosis assay in the HEK293, SKBR3 and MCF-7 cell lines at 20× magnification. Control = untreated (**A**) HEK293 (**B**) SKBR3 and (**C**) MCF-7 cells. FAuNP treated cells (**D**) HEK293 cells with Au-CS at the suboptimum ratio, (**E**) SKBR3 cells with Au-CS-FA-His at the supraoptimum ratio and (**F**) MCF-7 cells with Au-CS at the optimum ratio. Scale bar = 100 µm.

To determine whether the nanocomplexes induced any cell death by apoptosis or necrosis, a fluorescent assay using the acridine orange/ethidium bromide (AO/EB) dual stain was conducted. To correlate the results of the cytotoxicity assay, this assay was performed using the ratio of the nanocomplexes with the lowest cell viability, viz., suboptimum ratio for the Au-CS nanocomplexes in the HEK293 cells, the supraoptimum ratio for the Au-CS-FA-His nanocomplexes in the SKBR3 cells and the optimum ratio for the Au-CS nanocomplexes in the MCF-7 cells. The fluorescent images (Figure 8) and the calculated apoptotic indices (Table 3) revealed that, although the exposure of the nanocomplexes to the cells may have induced some apoptosis, the majority of cells present were viable (green), supporting the low cytotoxicity observed in the MTT assay.

### 3.7. Transfection Studies

**Figure 9:**
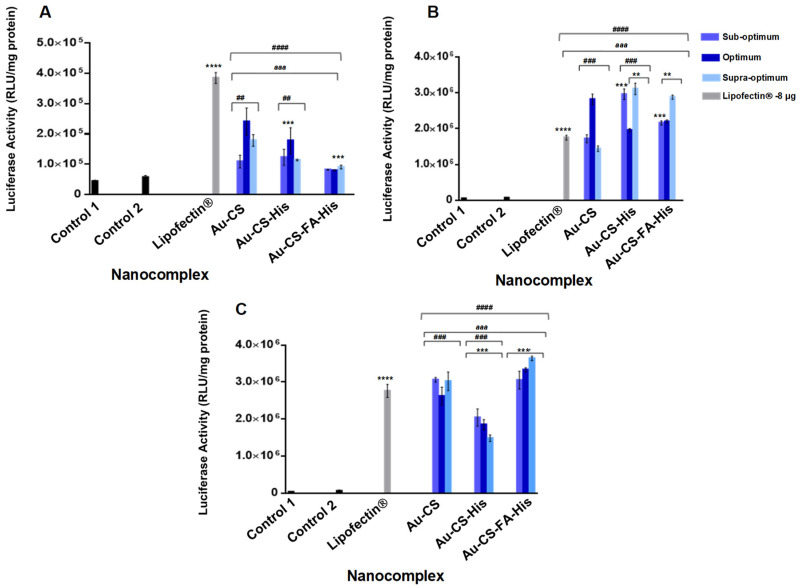
Luciferase activity in (**A**) HEK293, (**B**) SKBR3 and (**C**) MCF-7 cells. Data are represented as the mean ± SD (*n* = 3). Control 1: untreated cells and Control 2: naked pDNA-treated cells. Lipofectin^®^:pDNA complexes at the optimal gene expression were used as a positive control. ** *p* < 0.01 and *** *p* < 0.001 show statistical significance between the corresponding ratios of Au-CS-FA-His vs. Au-CS-His, **** *p* < 0.0001 vs. control 1, ^##^ *p* < 0.01 and ^###^ *p* < 0.001 show statistical significance between the corresponding ratios of Au-CS-His vs. Au-CS, ^aaa^ *p* < 0.001 shows statistical significance between the respective nanocomplexes vs. control 2 and ^####^ *p* < 0.0001 shows statistical significance between FAuNPs vs. Lipofectin^®^. The suboptimum, optimum and supraoptimum ratios are indicated in Table 1.

The transfection efficiency of the FAuNPs was evaluated using the luciferase reporter gene assay in the HEK293, MCF-7 and SKBR3 cells. Three controls were used and included cells in the absence of FAuNPs and pDNA (Control 1), cells treated with naked pDNA (Control 2) and cells treated with complexes formed with the commercial transfection reagent Lipofectin^®^. The results of the Lipofectin^®^:pDNA complexes in all the cell lines at their different ratios are presented in Supplementary Figure S5. The Lipofectin^®^:pDNA complex ratio that elicited the highest transfection in each cell line was plotted against the respective NPs in Figure 9.

All the nanocomplexes elicited transgene expression in the three cell lines, with the luciferase activity greater than that of the naked pDNA (Control 2). The control Lipofectin^®^, however, showed exceptionally high transfection in the HEK293 cells compared to the breast cancer cells, where the transgene expression was marginally lower than that of the FAuNP nanocomplexes. For the FAuNP nanocomplexes, luciferase activity was lowest in the HEK293 cells and ranged from 7.98 × 10^4^ to 2.41 × 10^5^ RLU/mg protein (Figure 9A). The conjugation of His to Au-CS produced a significant increase in the luciferase activity at the suboptimum and supraoptimum ratios (*p* < 0.001) in the SKBR3 cells (Figure 9B). Interestingly, this was not reflected in the other cell lines, suggesting that the use of His for enhanced transfection efficiency could depend on the cell type. The Au-CS-FA-His nanocomplex exhibited superior transfection efficiency over their untargeted nanocomplexes, with the highest transfection efficiency at all three ratios in the FR-overexpressing MCF-7 cell line (Figure 9C). These results suggest that cellular uptake of the Au-CS-FA-His nanocomplexes occurred via receptor-mediated endocytosis (RME).

### 3.8. Receptor Competition Assay

**Figure 10:**
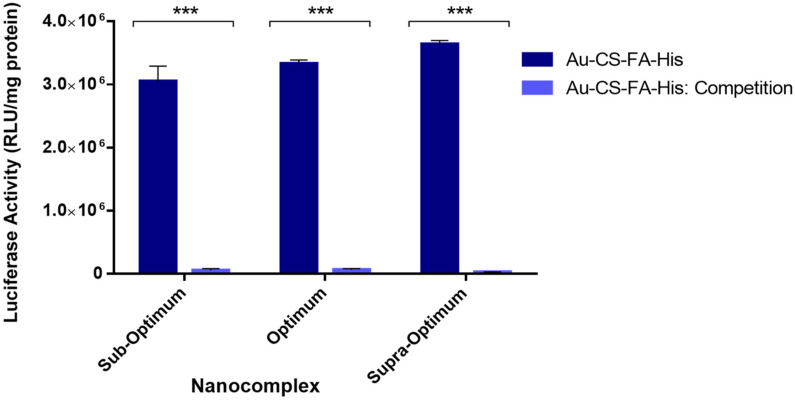
Competition binding assay of Au-CS-FA-His nanocomplexes in the MCF-7 cell lines. Data are represented as the mean ± SD (*n* = 3). *** *p* < 0.001 shows statistical significance between the corresponding ratios of Au-CS-FA-His vs. Au-CS-FA-His: Competition. The suboptimum, optimum and supraoptimum ratios are indicated in Table 1.

The receptor competition binding assay was carried out to confirm that cellular uptake of the Au-CS-FA-His nanocomplex occurred via RME and that the enhanced transfection efficiency of this nanocomplex in the MCF-7 cells was due to the presence of FA in the nanocomplex. The assay involved the prior flooding of the FR-positive MCF-7 cells with excess free FA, which would occupy the receptor sites on the cells and prevent the targeted nanocomplex from recognizing and binding to the FRs. Hence, this would reduce or prevent RME and lower the luciferase activity for the targeted nanocomplexes. Figure 10 depicts the results of the receptor competition binding assay in the MCF-7 cells.

The results indicate that the luciferase activity of Au-CS-FA-His was significantly reduced due to the blocking of the MCF-7 FR sites by the excess FA (*p* < 0.001). In the presence of the competitor, a 135-fold decrease in luciferase activity was observed at the supraoptimum ratio, followed by a 55-fold and 51-fold reduction at the suboptimum and optimum ratios, respectively. These results provide evidence that cellular uptake of the Au-CS-FA-His nanocomplexes in the MCF-7 cells occurred predominately via RME, consistent with previous reports where FA-conjugated vectors have shown a high affinity for FRs [59,60,61].

## 4. Discussion

Targeted gene delivery and endosomal escape are some of the challenges facing successful gene delivery. Hence, the formulation and identification of such modalities will form part of ongoing investigations in this area. Nonviral delivery vehicles such as AuNPs have emerged as strong contenders as they can be readily functionalized with targeting ligands and other endosomal-disrupting moieties. In this study, AuNPs were successfully synthesized using an adapted Turkevich method, allowing for greater control of the AuNP size and concentration [62,63]. The surface modification with CS, His and FA aimed to increase the stability of the AuNPs and enhance their potential as efficient gene delivery vehicles. In addition to providing stability in an aqueous medium, CS was included for its many cationic amine groups that can electrostatically bind to the negatively charged phosphate groups of the pDNA and hydroxyl groups, which are useful binding sites for many other molecules [33]. The medium molecular weight CS was chosen based on previous studies by the authors, which showed that this molecular weight and the chosen concentration was easy to conjugate to the AuNPs, in addition to producing stable NPs of favorable sizes [33,64,65]. The use of His was based on the need for the early endosomal escape of the gene cargo before it can be degraded. At the same time, FA was conjugated to the NP to facilitate active cell-specific targeting to tumor cells expressing the FR. The successful synthesis and functionalization of the AuNPs were confirmed visually by a color variation from the wine red of the AuNPs. UV–Vis spectroscopy produced an excitation peak of 521 nm for the AuNPs, while functionalization led to red shifts in the surface plasmon resonance similar to that previously reported [60]. FTIR spectroscopy further confirmed the presence of the specific functional group/s native to the NPs.

The sizes of the NPs and nanocomplexes obtained in this study can be considered favorable, since NPs ranging between 100 and 200 nm are regarded as optimal for gene delivery [66]. Functionalization of the AuNPs and their formation of nanocomplexes led to an increase in size, as expected and reported previously [33,60]. The stability and negative charge of the AuNPs are due to the citrate that acted as the stabilizing agent during synthesis and the anionic carboxyl groups present [67]. All the FAuNPs exhibited positive ζ potentials resulting from the conjugation of CS to the AuNPs, which provides a high cationic charge due to its many protonated amino groups [68]. It is important to note that the determined ζ potential is a more accurate indicator of colloidal stability than the surface charge [69]. As mentioned earlier, the zeta potentials obtained for the NPs and the nanocomplexes all fell within the range for NPs with good colloidal stability. This argues well for the use of these NPs in an in vivo system.

The FAuNPs were able to successfully bind the pDNA at varying ratios due to the presence of the different functionalities on the NPs. In the case of Au-CS-FA-His, the binding of the anionic carboxyl group of FA to the cationic amino group of CS reduced the number of positive charges available [70], resulting in more of the NP being required to bind the same amount of pDNA. Although His increased the number of positive charges, the pDNA interaction may have been weakened by the possible steric hindrance of His [71] and the lower pK_a_ of the α-amino group [72]. Furthermore, the low compaction of Au-CS-FA-His noted in the dye displacement assay can be attributed to the presence of FA, which partially shielded the positive charges [73]. In contrast, a good Au-CS compaction was expected and highlighted the ability of CS to bind efficiently and compact DNA. Interestingly, Au-CS-His had the strongest compaction, which could be attributed to the vast number of positive charges present due to CS and His on the NP. It is important to note that the endpoint determined in the EB intercalation assay may not directly correlate to that obtained in the band shift assay. The band shift assay identifies the point at which the negative charges of the pDNA is completely neutralized by the FAuNPs. In contrast, the EB intercalation assay purely assesses the degree of compaction of the pDNA by the FAuNPs.

Overall, the FAuNPs were observed to be able to protect the pDNA cargo from nuclease digestion. However, at the supraoptimum ratios of Au-CS and Au-CS-His, much of the pDNA was still bound to the FAuNPs and was retained in the wells, as evidenced by the bright fluorescence in the wells. This suggests that the strong interaction within the nanocomplex could not be completely broken down to allow for DNA release, despite the addition of sodium dodecyl sulphate. This detergent has been commonly used to release nucleic acids from its carrier molecule. A similar strong binding for AuNPs has been reported [60,74]. This correlates to the high compaction ability of Au-CS and Au-CS-His, as seen in the EB displacement assay.

The nanocomplexes were all well-tolerated in all the cell lines. Interestingly, the optimum ratio of Au-CS-His produced a significant increase in cell viability in the MCF-7 cells, which exceeded the control (100%). Since His is widely known to play a vital role in the growth and repair of tissue [75], it can be proposed that the conjugation of His to the FAuNPs is the reason for this promotion of cell growth. It is also important to note that the cytotoxicity of a protonated imidazole ring, such as in His, is considered less toxic than the protonated amine groups present in CS [76]. A similar increase was also noted for Au-CS-FA-His. This can further be explained by highlighting the importance of FA in cell growth, as FA is involved in the synthesis of purines and pyrimidines during the DNA cycle [77]. Overall, the cytotoxicity profiles of the nanocomplexes in all three cell lines suggest that these FAuNPs are relatively safe for gene delivery. The AO/EB apoptosis assay was used to determine whether cell death occurred by apoptosis (programmed cell death) or necrosis (premature cell death) by visualizing the changes in the cell fluorescence, shape and chromatin [78]. Acridine orange (AO) dye can penetrate viable and nonviable cells, staining the green nuclei. Since AO dye cannot differentiate between the cells, a mixture of AO and EB was used. EB solely penetrates dead cells, since their membrane has lost integrity, thus staining the nuclei red [79]. Overall, there was a good correlation between the MTT and the AO/EB apoptosis assays. A high cell viability was confirmed by the low apoptotic indices obtained and the predominance of viable (green) cells in the images (Figure 8). Furthermore, due to the absence of necrotic cells, it can be deduced that these nanocomplexes did not cause necrosis in any of the cell lines that were tested.

Transfection conducted using reporter pCMV-*Luc*-DNA expressing the luciferase gene was successful in all the cells, as evidenced by the high levels of measured luminescence against the controls and the Lipofectin^®^ reagent. This luciferase expression was notably higher in the two breast cancer cell lines than in the normal HEK293 cells and was in the order of MCF-7 > SKBR3 > HEK293. The Lipofectin^®^ reagent performed optimally in the HEK293 cells compared to the two breast cancer cell lines, with the luciferase activity higher that the other nanocomplexes. This reagent is known to successfully transfect a wide range of cells, including those investigated in this study. Overall, the FAuNP nanocomplexes, except Au-CS at the suboptimal and supraoptimal ratios, elicited slightly higher transfection activities in the SKBR3 cells than Lipofectin^®^. In the MCF-7 cells, only Au-CS and Au-CS-FA-His had higher transgene expressions than the Lipofectin^®^ control. Furthermore, the FA-targeted nanocomplexes (Au-Cs-FA-His) were the best-performing nanocomplexes against the Lipofectin^®^ control. It is important to note that the amount of pDNA (1 µg) used for the Lipofectin^®^ reagent was more than twice that used for the FAuNPs. Similar cell-specific results with Lipofectin^®^ were observed despite the higher amount of pDNA used [9]. The increase in luciferase activity of the Au-CS-His nanocomplexes at the suboptimum and supraoptimum ratios in the SKBR3 cells can be attributed to the primary function of His, which is to enhance endosomal escape through its proton sponge effect. Its protonated imidazole side chain ensures a high buffering capacity, allowing the internalized cargo to escape the endosome and avoid degradation [80]. Interestingly, this was not reflected in the other cell lines, suggesting the use of His for enhanced transfection efficiency could depend on the cell type, cell surface properties and receptors and the type of biomolecule appended to the NP [81]. Overall, the Au-CS-FA-His nanocomplexes produced significantly higher transgene expressions exceeding those of the untargeted nanocomplexes, especially in the FR-overexpressing MCF-7 cells, suggesting that the Au-CS-FA-His nanocomplexes were taken up by RME. The presence of His in the preparation may have also influenced the transgene expression, providing some synergistic support in the overall transfection process. Based on the different results in the SKBR3 and MCF-7 cells, it can be inferred that the His and FA concentrations may need to be optimized further for application in these cells. Since there are more FRs on the surfaces of the MCF-7 cells than on the SKBR3 cells [81], it is expected that RME may be more pronounced in these cells. In comparison, the HEK293 cells are considered FR-negative, producing a reduced luciferase expression [82,83]. Based on the transgene activity and the FR distribution in the cells tested, the MCF-7 cells were chosen for the receptor competition study. Similar reports have shown enhanced specificity in MCF-7 cells due to FR-mediated endocytosis [60,61].

Overall, this proof of principle study has confirmed that these nanocomplexes can safely and efficiently deliver a reported gene in vitro. In-depth studies employing a therapeutic gene are required to better understand these nanocomplexes for future in vivo applications. The theranostic potential, especially of the Au-CS-His and Au-CS-FA-His nanocomplexes, can be further explored in gene therapy and diagnosis in breast cancer cells.

## 5. Conclusions

This study aimed to manipulate the favorable properties and parameters of AuNPs to ensure that the pCMV-*Luc*-DNA reporter gene was both safe and efficient in its transfection. The FAuNPs and their nanocomplexes exhibited nanoscale sizes, high stability, low cellular toxicity, apoptotic indices and high transgene activity in the MCF-7 and SKBR3 cells compared to the noncancer HEK293 cells. Importantly, they also produced slightly higher transgene expression in the breast cancer cells compared to the Lipofectin^®^ control. This supports the use of these FAuNPs as potential gene delivery vehicles for breast cancer therapy, as they are stable, biocompatible and exhibit cell specificity. The transfection efficiency in the SKBR3 cells was enhanced by the inclusion of His in the formulation. However, this was not as prominent in the MCF-7 and HEK293 cell lines, which suggests that the efficiency of His-containing NPs can be cell type-specific and warrants further investigation into their use for the possible treatment of HER2-overexpressing cancers. Furthermore, the addition of His and FA increased the in vitro efficiency of the targeted nanocomplex, with superior transgene expression observed in the FR-rich MCF-7 cells. This synergistic advantage of FA and His needs to be further investigated by optimizing the ratios of these two biomolecules in NP formulation. Overall, these FAuNPs have effectively demonstrated their potential as nonviral gene delivery vehicles. This proof of principle study provides a possible foundation to address the shortcomings and challenges experienced in nonviral gene delivery, especially in breast cancer treatment.

## Figures and Tables

**Table 1:** Suboptimum, optimum and supraoptimum FAuNP:pDNA (*w*/*w)* ratios that were obtained from the band shift assay.

Nanocomplexes	Suboptimum	Optimum	Supraoptimum
Au-CS: pDNA	1.5:1	2:1	2.5:1
Au-CS-His: pDNA	2.8:1	3.2:1	3.6:1
Au-CS-FA-His: pDNA	4.5:1	5:1	5.5:1

**Table 2:** Hydrodynamic size, zeta (ζ) potential and polydispersity index (PDI) values of AuNPs, FAuNPs and their respective nanocomplexes with pDNA.

	Nanoparticles			Nanocomplexes			
	Size (nm ± SD)	ζ Potential (mV ± SD)	PDI	pDNA:NP Ratio (*^w^/_w_*)	Size (nm ± SD)	ζ Potential (mV ± SD)	PDI
AuNP	79.9 ± 12.0	−24.9 ± 0.2	0.023	-	-	-	-
Au-CS	116.5 ± 11.9	35.7 ± 0.2	0.011	1:2	129.7 ± 44.6	−21.5 ± 2.1	0.118
Au-CS-His	121.5 ± 4.0	38.0 ± 1.6	0.0011	1:3.2	133.1 ± 13.5	−23.4 ± 0.7	0.01
Au-CS-FA-His	122.2 ± 21.1	28.5 ± 3.3	0.029	1:5	126.3 ± 5.0	−34.2 ± 0.3	0.0016

**Table 3:** Apoptotic indices of the nanocomplexes.

Cell Lines	Apoptotic Index		
	Au-CS Suboptimum	Au-CS-FA-His Supraoptimum	Au-CS Optimum
HEK293	0.032	-	-
SKBR3	-	0.036	-
MCF-7	-	-	0.026

## Data Availability

The data and contributions presented in the study are included in the article and supplementary file. Further inquiries can be directed to the corresponding author.

## Supplementary Materials

The following are available online at https://www.mdpi.com/article/10.3390/pharmaceutics14010053/s1: Figure S1: FTIR spectrum of chitosan. Figure S2: FTIR spectrum of histidine. Figure S3: FTIR spectrum of folate. Figure S4: FTIR spectrum of CS-FA. Figure S5: Transfection studies of Lipofectin^®^:pCMV- *Luc* plasmid DNA. Data are presented as the means ± SD (*n* = 3). Statistical analysis among the mean values was performed using one-way ANOVA followed by Dunnet’s multiple comparisons test vs. control 1. **** *p* < 0.0001.
