# Supplementary material for: Histidine-Tagged Folate-Targeted Gold Nanoparticles for Enhanced Transgene Expression in Breast Cancer Cells In Vitro
Source: Pharmaceutics. 2021 Dec 27;14(1):53. doi: 10.3390/pharmaceutics14010053 (PMC8781941; doi:10.3390/pharmaceutics14010053)
Supplement: Supplementary file 1 [file pharmaceutics-14-00053-s001.zip › pharmaceutics-1491593-supplementary material-updated.pdf]

pharmaceutics

MDPI

Supplementary Materials: Histidine-tagged Folate-Targeted Gold Nanoparticles for enhanced transgene expression in Breast Cancer Cells in Vitro

Calrin Joseph, Aliscia Daniels, Sooboo Singh and Moganavelli Singh

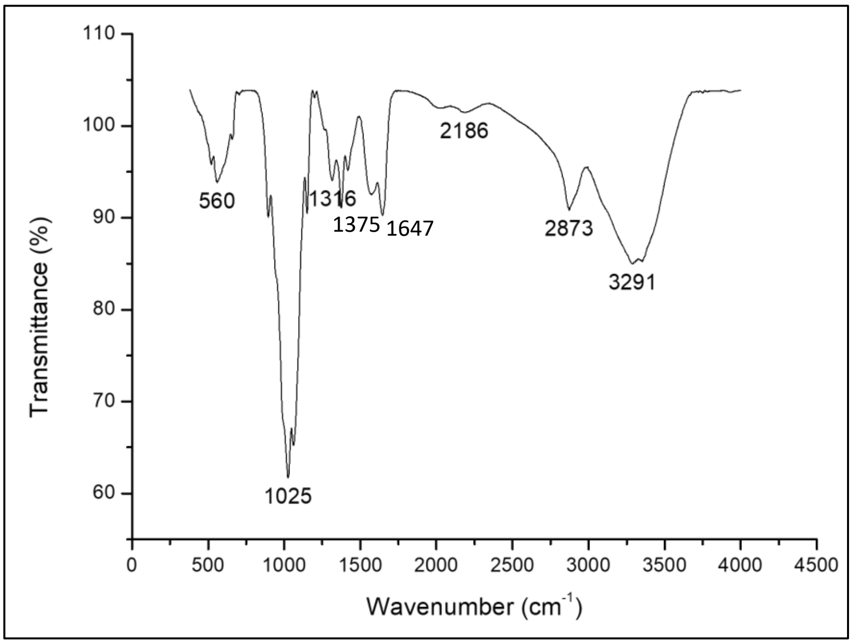

Figure S1. FTIR spectrum of Chitosan.

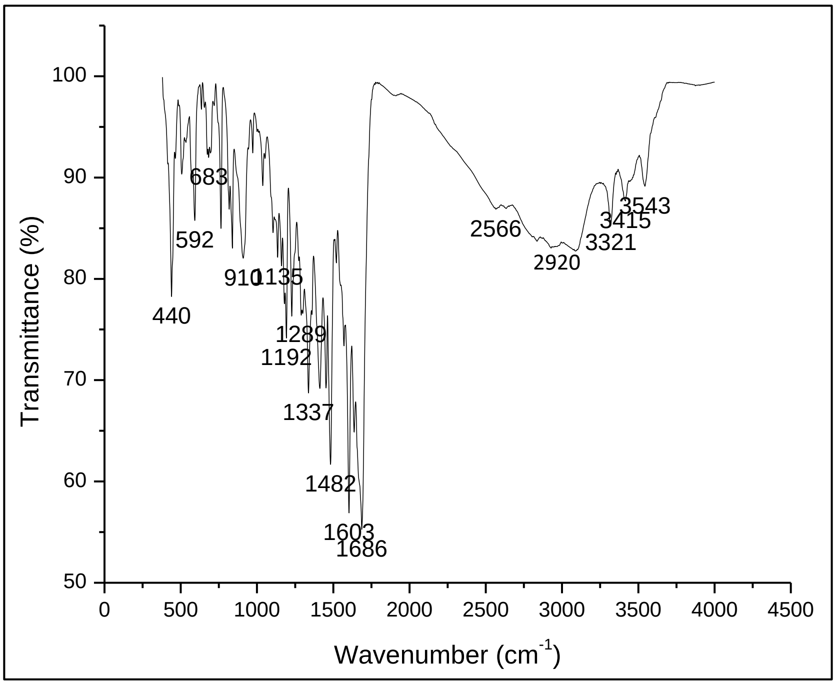

Figure S2. FTIR spectrum of Histidine.

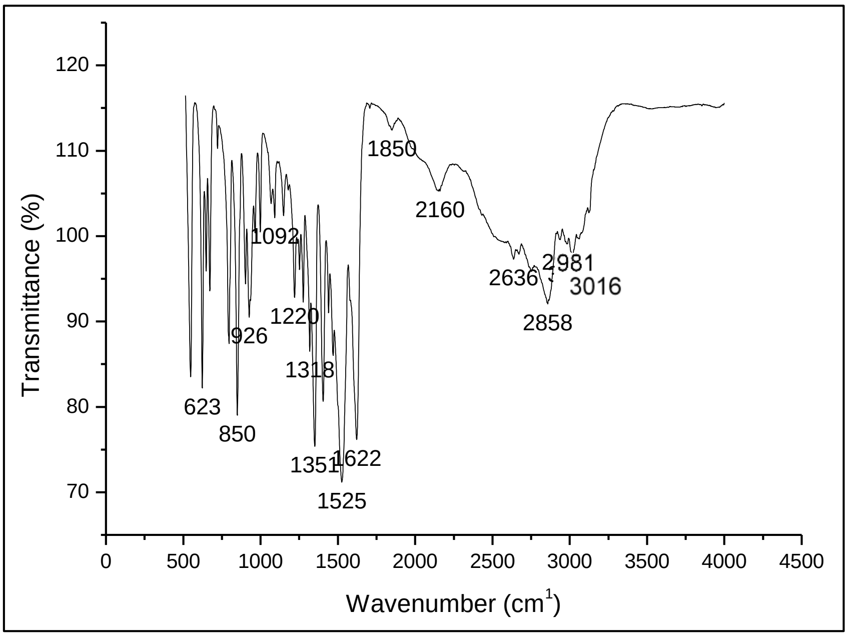

Figure S3. FTIR spectrum of Folate.

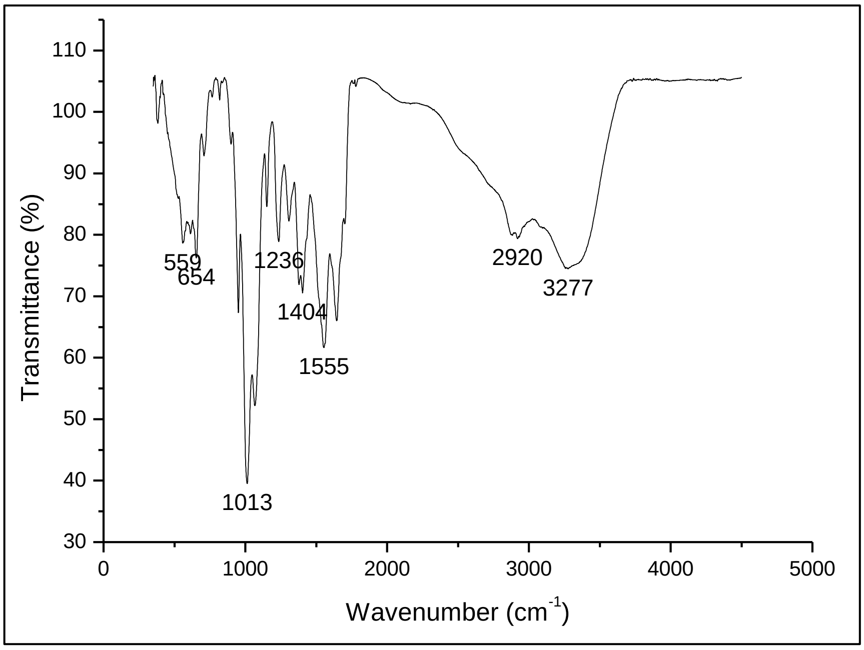

Figure S4. FTIR spectrum of CS-FA.

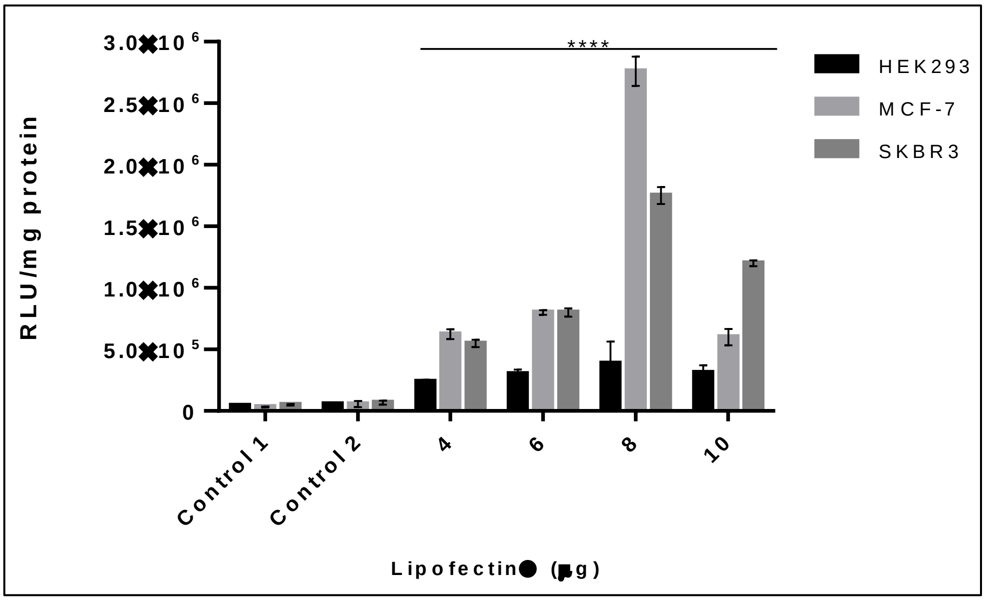

Figure S5. Transfection studies of Lipofectin®: pCMV- luc plasmid DNA. Data are presented as means ± SD (n = 3). Statistical analysis among mean values was performed using one-way ANOVA followed by Dunnets multiple comparisons test vs control 1. **** $p < 0.0001$.
